# Motivational Profiles of High School Physical Education Students: The Role of Controlling Teacher Behavior

**DOI:** 10.3390/ijerph16101714

**Published:** 2019-05-16

**Authors:** Elisa Huéscar Hernández, Juan Antonio Moreno-Murcia, Lorena Ruíz González, Jaime León González

**Affiliations:** 1 Department of Health Psychology, Miguel Hernández University of Elche, Avda. Universidad, s/n, 30202 Elche, Spain; 2 Sports Research Center, Miguel Hernández University of Elche, Avda. de la Universidad, s/n, 30202 Elche, Spain; j.moreno@umh.es; 3 Department of Social Psychology, University of Málaga; Avda. Cervantes, 2, 29071 Málaga, Spain; klera_lorka@hotmail.com; 4 Department of Education, University of Las Palmas de Gran Canaria, C/Santa Juana de Arco, 1, 35004 Las Palmas de Gran Canaria, Spain; jaime.leon@ulpgc.es

**Keywords:** teaching style, motivation, self-determination theory, secondary education

## Abstract

*(1) Background*: The purpose of this study was to identify distinct motivational profiles in high school Physical Education students. These motivational profiles were examined in relation to controlling teacher behaviors, as well as to various psychological correlates including the perceived importance of physical activity to the student, student intentions to be physically active, psychological need satisfaction and current physical activity levels. *(2) Methods:* 416 high school Physical Education students comprised the sample. *(3) Results*: Cluster analysis and additional multivariate analyses revealed two motivational profiles, *Wilk’s Λ* = 0.56, *F* (7, 431) = 45.50, *p* < 0.01. The “Self-Determined” profile was characterized by high levels of self-determined motivation; high levels of competence, autonomy and relatedness; importance of physical activity; stronger intentions to engage in physical activity; and greater current actual physical activity involvement. The “Less Self-Determined” profile was associated with the perception of controlling teacher behaviors, and with greater external regulation and amotivation. *(4) Conclusions*: These findings help to provide new insights into the explanation of student motivation in Physical Education and the design of intervention programs.

## 1. Introduction

Regular participation in physical activity is known to have resulting benefits for physical health and psychological outcomes [1] and also contributes to improved academic performance in students [2]. However, about 32.8% of the population older than 15 years of age engages in very low levels of physical activity, whereas another 12.3% engages in no physical activity whatsoever [3]. Research indicates that there is a marked decline in rates of physical activity between the ages of 12 and 18 years [4], and this age range thus represents an important window of opportunity to attempt to influence physical activity orientations in young people. Physical Education classes should be integral to these efforts as they constitute essential learning opportunities for promoting healthy lifestyles in adolescents. A primary challenge is to attempt to strengthen student motivation for continued physical activity engagement outside of the classroom [5]. Consequently, the instructional style of teachers is important to consider, as it affects their students’ subsequent motivation and behavior outside the walls of the classroom.

From the perspective of Deci and Ryan’s [6] Self-Determination Theory (SDT), motivation is considered in relation to the extent to which it is self-controlled. Motivation is an aspect of great relevance because it orients the actions to become a central element with respect to the person’s objectives. The SDT perspective is intended to explain the manner in which motivated behavior can be manifested across a continuum in which any individual’s motives reflect their feelings of self-determination. The intrinsic motivation is the most self-determined, and is at the opposite extreme from amotivation. Amotivation lies at one end of the continuum and represents the absence of motivation. The four manifestations of extrinsic motivation are external regulation (behavior is performed to satisfy an external demand or the existence of prizes or rewards), introjected regulation (behavior is performed to gain appreciation or to avoid rejection and feelings of guilt or shame), identified regulation (behavior is highly valued and the person judges it as important) and integrated regulation (behavior is carried out freely and several identifications are assimilated and organized hierarchically), with each form representing an increasingly self-determined form of motivation. Teacher behavior is influential in feelings of self-determination because teachers who provide autonomy support for their students can strengthen their pupils’ positive intentions, preferences and values to participate voluntarily in an activity [7]. An autonomy-supportive teaching style can serve to strengthen the motivational resources of the student and, in turn, is associated with adaptive consequences in the learning domain at the behavioral and emotional levels [8] and is positively related to intrinsic motivation [9].

The nature of the student-teacher interaction can contribute to the motivational orientation demonstrated by the student. Unfavorable teaching behaviors can negatively impact the student’s perceptions and feelings, as well as their subsequent behavior. A controlling teaching style, in particular, can negatively affect student motivation and limit the positive experiences afforded in the learning environment [10], although there have been relatively few studies that have been conducted that have examined teacher influence [11,12]. Thus, the study of motivation is important in the field of physical activity and education because it is at the heart of biological, cognitive and social regulation.

Various studies have attempted to identify distinct motivational profiles related to physical activity in both Physical Education and extracurricular contexts [13], as the ability to identify such profiles would contribute to the design of pedagogical interventions for those learners with limited self-determined motivation. Research indicates that more self-determined individuals characteristically feel greater basic psychological need satisfaction [14]. Basic Psychological Needs Theory (BPNT) [15] is part of SDT and specifies the innate and essential psychological aspects for continued psychological growth, integrity and well-being. These needs are the psychological mediators that will influence motivation. BPNT is grounded in the belief that the satisfaction of the essential needs of autonomy (reflecting perceived choice and self-regulation of one’s own actions); competence (feelings of efficacy and confidence regarding one’s ability to complete a task); and relatedness (presence of positive relationships with others and feelings of integration within the group), will contribute to intrinsic motivation. Teachers can contribute to the satisfaction of these basic psychological needs to the extent that they engage in appropriate interpersonal interactions with their students, particularly by allowing greater student autonomy, which contributes to persistence and which can strengthen intentions to be physically active in the future [16]. For example, in order for the teacher to satisfy the student’s psychological need for autonomy, the teacher could give the initiative to the student. In order to satisfy the psychological need of the student of competence, the teacher can explain the structure of the task with respect to the class, and to satisfy the psychological need of relationship, the teacher can listen to the students with an active and positive attitude.

The high school years represent an essential phase in the development of favorable physical activity orientations and practices, and it is a time to help students learn of the negative consequences associated with physically inactive lifestyles. The purpose of this study was to identify distinct motivational profiles in high school Physical Education students to examine the relationship of these with students’ perceptions of controlling teacher behaviors, psychological need satisfaction, perceived importance of Physical Education, physical activity intentions and actual physical activity levels. The purpose of this analysis was to identify homogeneous groups or clusters based on their common characteristics. It was hypothesized that more self-determined motivational profiles would reflect greater psychological need satisfaction as well as greater perceived importance of Physical Education. Students with more self-determined profiles were also anticipated to possess stronger intentions to be physically active and to engage in greater amounts of physical activity. It was expected that less self-determined motivational profiles would be associated with student perceptions of a controlling teaching style. 

## 2. Materials and Methods

### 2.1. Ethics Statement

This study has been approved by the Research Ethics Committee of Universidad Miguel Hernández de Elche (Elche, Spain) (DPS.JMM.01.17) and meets all ethical and legal standards that are applicable to the research of this survey modality.

### 2.2. Participants

The sample was comprised of 416 students, of whom 229 were male and 187 were female between the ages of 16 and 18 years of age (*M* = 16.71 years., *SD* = 0.73 years.). The participants were students at a public high school. 

### 2.3. Measures

#### 2.3.1. Controlling Teaching Behaviors

To assess controlling teaching style, a modified version of the Controlling Coach Behavior Scale (CCBS) was used. This instrument had been developed by Bartholomew, Ntoumanis and Thøgersen-Ntoumani [17], and was validated in the Spanish cultural physical education context by Castillo et al. [18]. The instrument assesses controlling behaviors during the completion of Physical Education tasks by students. The measure consists of 15 items, uses the stem phrase, “In reference to my Physical Education teacher…” and responses fit into one of four subscales. These subscales include: controlling through the use of rewards (e.g., “My teacher tries to motivate me by promising to reward me if I do well”); negative consequences (“My teacher is less accepting of me if I have disappointed him/her”); use of intimidation (“My teacher shouts at me in front of the others to make me do certain things”); and excessive personal control (“My teacher expects my whole life to center on my Physical Education participation”). The questions were responded to through a Likert scale format ranging from “1” (“totally disagree”) to “7” (totally agree). The internal consistency estimates of the four subscales were 0.69, 0.73, 0.85 and 0.78 respectively. The overall Cronbach estimate of internal consistency for the entire scale was 0.94.

#### 2.3.2. Basic Psychological Needs

The Psychological Need Satisfaction in Exercise Scale (PNSE), which was developed by Wilson, Rogers, Rodgers and Wild [19] and validated in the Spanish context by Moreno-Murcia, Marzo, Martínez-Galindo and Conte [20], was employed to assess the three essential psychological needs proposed by SDT with specific reference to the Physical Education context. The scale consists of 18 items grouped into three separate subscales with each subscale consisting of six items. The stem phrase for each question was, “In classes of Physical Education” and sample items for the subscales included, “I believe that I can complete personal challenges” (competency); “I feel that I can complete tasks in the way that I prefer” (autonomy); and “I feel that I get along well with others when I engage in the activities with others” (relatedness). The response choices range from “1” (“False”) to “6” (“True”). The Cronbach internal consistency values for the three subscales were 0.91, 0.84 and 0.77, respectively.

#### 2.3.3. Behavioral Regulation in Sport

The Behavioral Regulation in Sport Questionnaire (BRSQ), developed by Lonsdale, Hodge and Rose [21] and validated in the Spanish cultural context by Moreno-Murcia et al. [20], was adapted for use in the Physical Education context. Intrinsic motivation was assessed through a subscale of the BRSQ. 

This instrument assesses different types of motivation in physical activity and consists of 36 items grouped along nine dimensions. The dimensions include general intrinsic motivation (e.g., “Because I enjoy it”); for knowledge (e.g., “For the pleasure that I gain understanding more about this activity”); for stimulation (e.g., “Because I enjoy the intense stimulation that I feel when I engage in this physical activity”); toward achievement (e.g., “Because I enjoy it when I reach long-term goals”); integrated regulation (e.g., “Because it is part of who I am”); identified regulation (e.g., “Because the benefits of physical activity are important to me”); introjected regulation (e.g., “Because I would feel ashamed if I quit”); external regulation (e.g., “Because if I don’t other people will not be pleased”); and amotivation (e.g., “I question why I am putting myself through this”). The common stem phrase for each of the questions is, “I participate in my physical activity…”. The response options ranged from “1” (“very false”) to “7” (“very true”) in a Likert-type format. The internal consistency estimates for the subscales ranged from 0.81 to 0.91.

#### 2.3.4. Importance of Physical Education 

A three-item scale was used to measure students’ perceptions of the importance and utility value of Physical Education. The measure was developed by Moreno, González-Cutre and Ruíz [22], and includes sample statements such as, “I think it is important to take Physical Education classes”. The response format ranges from “totally agree” to “totally disagree” on a 4-point Likert-type scale. The Cronbach alpha internal consistency value of the scale was 0.76.

#### 2.3.5. Physical Activity Intentions 

The Intention to be Physically Active Scale [23] was employed as this scale has been translated and adapted from the original version by Moreno, Moreno and Cervelló [24]. The measure consists of five items that assess individuals’ intentions to be physically active using the stem, “With respect to your intention to practice physical activity or sport…”. A sample statement is, “After finishing my school day I like to be physically active”. The response format conforms to a five-point Likert-type structure with endpoints of “totally disagree” and “totally agree,” with responses that indicate a stronger desire to be physically active receiving higher values. The Cronbach alpha estimate of internal consistency was 0.81 for this scale.

#### 2.3.6. Habitual Physical Activity 

The Spanish language version [25] of the Habitual Physical Activity Questionnaire [26] was used to assess the free-time physical activity behavior of the participants. Four questions comprise the scale, and the first question refers to the type of sport or physical activity that the individual engages in and the weekly and monthly frequency in which the individual participates in the activity. An equation is used to reflect the intensity, time spent and frequency of physical activity that typically occurs each month for the two most common forms of activity. The equation is simply: Type 1 activity (intensity × time × monthly frequency) + Type 2 activity (intensity × time × monthly frequency). Types of physical activity receive different weightings relative to the demands of the activity [27]. The three remaining statements assess the amount of physical activity during the individual’s free time (e.g., “During my free time I engage in sport and physical activity”) utilizing a range from “1” (“Never”) to “5” (“Very frequently”), and the mean values are generated by calculating an overall scale mean.

### 2.4. Procedure

The directors of various high schools were contacted to inform them of the purpose of the investigation and to request their involvement. Parental informed consent was requested and necessary before the participants could participate. The students also were informed about the general purpose of the study and also provided written consent. The questionnaires were completed by the students in their classes of physical education under the supervision of the principal investigator who also addressed any questions or issues that arose. The questionnaires were completed by the students in a quiet atmosphere that enabled them to concentrate on the task and averaged about twenty minutes to complete. Student participation was entirely voluntary and individuals did not disclose any personal information that could be associated with their identity.

### 2.5. Data Analysis

Descriptive statistics were calculated for all variables in the study, including means and standard deviations (Table 1). An analysis of bivariate correlations was performed for the study variables (Table 2). The subsequent focus was upon identifying distinct motivational profiles within the overall sample. A hierarchical cluster analysis using Ward’s method was conducted with the motivational variables from the BRSQ to help identify these motivational profiles. Subsequently, a confirmatory solution was attempted using a K means agglomerative method with a second sample. Finally, a hierarchical cluster analysis using Ward’s method was performed with the entire sample. To examine the characteristics of each motivational profile in relation to teacher controlling behaviors, the basic needs satisfaction variables, the perceived importance of physical education, the intention to be physical active and actual physical activity, a multivariate analysis of variance (MANOVA) was conducted. All analyses were conducted using the SPSS 25.0 statistical package (SPSS Inc., Chicago, IL, USA).

## 3. Results

### 3.1. Descriptive Statistics

A mean score of 2.61 was reported by the students relative to the extent of perceived control by their teachers. With regard to the basic psychological needs, students scored highest on competence, followed by autonomy and relatedness. In terms of type of motivation, means for intrinsic motivation were the highest and amotivation was the lowest. The mean score obtained for the importance of physical education was 2.77, intention to be physically active had a mean value of 3.70 and habitual physical activity reached a mean of 9.85.

Significant correlations emerged among key variables in the study (Table 2). Most notably, controlling teaching style was negatively and significantly related to general intrinsic motivation (*r* = −0.16). Nonetheless, a controlling teaching style was positively and significantly correlated with introjected regulation (*r* = 0.35), external regulation (*r* = 0.45) and amotivation (*r* = 0.40). In turn, external regulation was negatively related with competence (*r* = −0.12) and general intrinsic motivation (*r* = −0.15). In addition, amotivation was significantly and negatively related with motivation toward knowledge (*r* = −0.16), stimulation (*r* = −0.18), achievement (*r* = −0.20), integrated regulation (*r* = −0.10), identified regulation (*r* = −0.16), intentions to be physically active (*r* = −0.20) and level of habitual physical activity (*r* = −0.10).

### 3.2. Cluster Analysis

The cluster analysis was conducted in accordance with a sequence of stages recommended by Hair, Anderson, Tatham and Black [28]. Individual cases were examined for missing data and such cases were excluded from further analysis in the event of incomplete data. The overall sample was then split into two separate samples through randomization to yield two samples with 208 participants in each sample. The univariate distribution across all of the variables was then examined for violations of normality.

**Figure 1:**
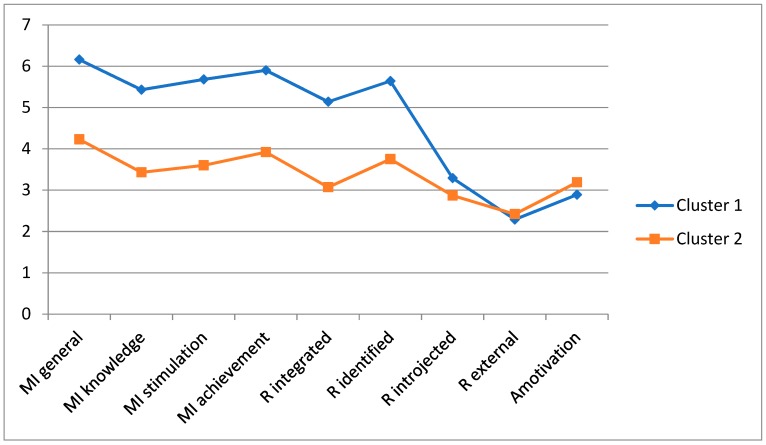
Analysis of Hierarchical Conglomerates with Ward Method in Sample 1. Note: MI = Intrinsic motivation; R = Regulation; Cluster 1 = Self-Determined; Cluster 2 = Less Self-Determined.

To differentiate resultant clusters in Sample 1, a hierarchical agglomerative approach was taken using Ward’s method. The decision about the number of clusters was based on the relative increase in agglomerative coefficients at each increase in potential cluster number. In accordance with Norusis [29], smaller coefficient values reflect a high level of homogeneity among the individual members of a cluster whereas larger coefficient values reflect greater differences among the individuals comprising the cluster. The resultant dendogram suggested the presence of two unique clusters (Table 3). Cluster 1 corresponded to the profile of “Self-Determined,” as the individuals within this cluster had relatively high scores on self-determined motivation (General Intrinsic Motivation and Motivation Toward Achievement, and very low levels of introjected regulation, external regulation and amotivation (Figure 1)). Cluster 2 was labeled “Less Self-Determined,” with lower levels of all types of intrinsic motivation, especially integrated regulation, and slightly elevated levels of external regulation and amotivation in relation to Cluster 1.

**Figure 2:**
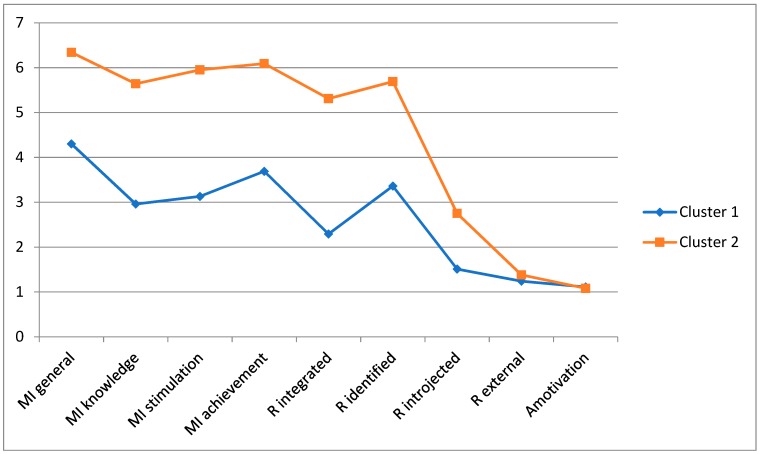
Analysis of average K-Clusters in Sample 2. Cluster 1 = Less Self-Determined; Cluster 2 = Self-Determined. Note: MI = Intrinsic motivation; R = Regulation.

To determine the motivational profiles of the individuals represented in Sample 2, a K-means analysis was similarly conducted (Figure 2). From this analysis, two profiles emerged. Cluster 1 demonstrated the profile of “Less Self-Determined,” with low levels on each type of self-determined motivation and very low levels of introjected regulation, external regulation and amotivation with values very similar to those of their counterparts in the first sample. Cluster 2 reflected a “Self-Determined” pattern, with very high values of self-determined motivation and very low introjected regulation, external regulation and amotivation. 

**Figure 3:**
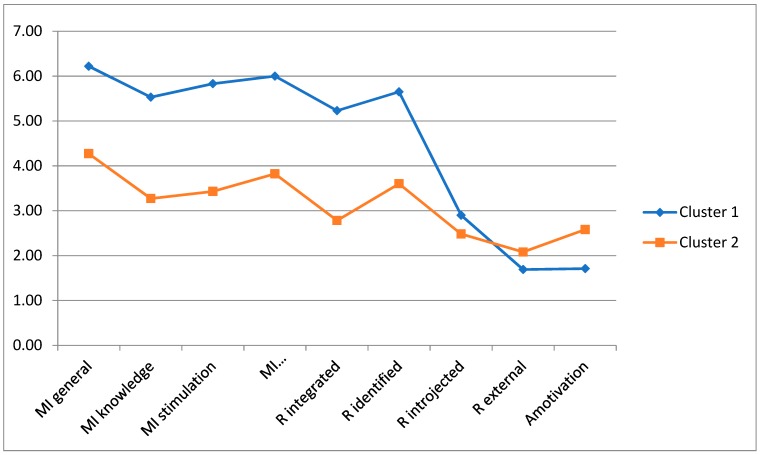
Analysis of Hierarchical Conglomerates with Ward Method in the Total Sample. Note: MI = Intrinsic motivation; R = Regulation; Cluster 1 = Self-Determined; Cluster 2 = Less Self-Determined.

Subsequently, a hierarchical agglomerative analysis was conducted using Ward’s method with the entire sample and two profiles once again emerged (Figure 3). One profile was labeled “Self-Determined” (Cluster 1), with high levels of self-determined motivation and low levels of introjected regulation, external regulation and amotivation. A second profile was labeled “Less Self-Determined” (Cluster 2), with lower values on self-determined forms of motivation and higher levels of external regulation and amotivation than the first cluster.

### 3.3. Multivariate Analysis

In order to examine the characteristics of each motivational profile in relation to the set of variables, a MANOVA was conducted with the sample as a whole. Accordingly, each cluster served as an independent variable, with controlling teaching style, basic need satisfaction, perceived importance of Physical education, intention to be physically active and level of actual physical activity serving as the dependent variables (Table 4). 

The results revealed significant differences between the “Self-Determined” cluster and the “Less Self-Determined” cluster in perceptions of controlling teacher behaviors, *Wilk’s Λ* = 0.56, *F* (7, 431) = 45.50, *p* < 0.01, and favored the “Less Self-Determined” cluster, *F* (1, 414) = 5.22, *p* < 0.05, ƞ^2^ = 0.01. The other group differences favored the “Self-Determined” cluster as these individuals were higher in Competence, *F* (1, 414) = 130.35, *p* < 0.01, ƞ^2^ = 0.24; autonomy, *F* (1, 414) = 72.21, *p* < 0.01, ƞ^2^ = 0.15; relatedness, *F* (1, 414) = 51.67, *p* < 0.01, ƞ^2^ = 0.11; perceived importance of physical activity, *F* (1, 414) = 72.21, *p* < 0.01, ƞ^2^ = 0.15; intention to be physically active, *F* (1, 414) = 249.09, *p* < 0.01, ƞ^2^ = 0.38; and level of habitual physical activity, *F* (1, 414) = 93.31, *p* < 0.01, ƞ^2^ = 0.19.

## 4. Discussion

Regular participation in physical activity is known to have resulting benefits for physical health and psychological outcomes and also contributes to improved academic performance in students. The primary purpose of this study was to identify motivational profiles of adolescent students in classes of Physical Education and to explore underlying differences in relation to controlling teaching style, basic psychological need satisfaction, perceived importance of Physical Education, intentions to be physically active and levels of actual physical activity behavior. Expectations were supported as the “Less Self-Determined” profile was associated with a controlling teaching style and the “Self-Determined” profile was associated with the remaining psychological variables.

The “Self-Determined” profile that was revealed through the cluster analysis was comprised of students with high levels of internal and identified regulation (intrinsic regulation), and low levels of external regulation and amotivation. The students represented by this cluster had stronger motives to engage in sport for the enjoyment and perceived benefits of the activity. In contrast, the “Less Self-Determined” profile had higher levels of external regulation and amotivation, as well as lower identified and intrinsic regulation. The students represented by this cluster reflected involvement that was characterized by the lack of self-determination motivation. Previous research has also encountered these two profiles in the educational context [30].

The students who were represented by the “Self-Determined” profile reported the highest level of satisfaction of their basic psychological needs, which is consistent with theoretical expectations [31]. Other desirable characteristics of this profile included a more favorable perception of the benefits of Physical Education, a stronger intention to be physically activity and greater actual physical activity involvement. These findings are consistent with those obtained by others studies [23,32].

The knowledge gained in this study is relevant to efforts designed to increase involvement in physical activity during adolescence, which is a critical phase of the lifespan for the establishment of favorable physical activity orientations. It has been argued that the accumulation of negative physical activity experiences can lead to habitual physical inactivity [33]. As such, the school environment presents an ideal context within which to attempt to develop a more active lifestyle and to reduce the tendency toward sedentary behavior [16]. As such, appropriate Physical Education experiences can strengthen the desire to be physically active [34,35].

The findings from this study provide a contribution to knowledge with regard to the social precursors of motivational expression in students. Students who feel more autonomous in the Physical Education context demonstrate a correspondingly stronger desire to be physically active. Previous research that has examined the application of Self-Determination Theory to the Physical Education setting has similarly found that student feelings of autonomy are instrumental in contributing to motivation [7,8,35]. At the same time, recent studies have revealed that maladaptive student orientations characterized by low self-determined motivation are tied to reports of controlling teacher behaviors [10]. In contrast, Physical Education students may perceive teacher autonomy support through a positive feedback cycle when students demonstrate knowledge gains and receive positive verbal and nonverbal feedback from teachers [36,37]. Thus, this study has two main educational implications. First, the theoretical framework of the SDT encourages teachers to move away from consistent controlling interactional styles where the teacher imposes on students their way of thinking, feeling and acting, due to the maladaptive consequences with which it is related. Secondly, this aspect related to the situational context of the class also seems to guarantee improvements in other aspects of student life, such as maintaining an active lifestyle.

With regard to the limitations of this study, it is important to note that a correlational design has been employed, which limits our ability to infer cause and effect, and the findings cannot necessarily be generalized beyond the present sample. Longitudinal designs are suggested for future research that capture the interactive effects of behavioral, affective and cognitive influences on motivation and behavior over time. In addition, it would be worthwhile to investigate the influence of gender, interactional style of the student and the influence of other social agents, such as family and peers in the motivational process.

## 5. Conclusions

In closing, this study has shown that students with a less self-determined profile perceive teachers with a controlling interpersonal style and they show worse motivational results, while the most self-determined ones are related to adaptive results on a physical, cognitive and emotional level. This provides new perspectives on how knowledge about relevant student motivational characteristics can facilitate the design of future physical activity promotion efforts in the school environment. The results can be useful for teachers to be able to regulate the insight of their motivational style towards their students by approaching a style of autonomy support for the benefits for the students that have been demonstrated.

## Figures and Tables

**Table 1:** Descriptive statistics among study variables.

Variables	Dimension	*M*	*SD*	*R*
Controlling Teaching Behavior	Teach Control	2.61	1.19	1–7
Basic Psychological Needs	Competence	4.35	1.18	1–6
	Autonomy	4.01	1.08	1–6
	Relatedness	3.70	1.03	1–6
Behavioral regulation in sport	IM general	5.39	1.45	1–7
	IM knowledge	4.57	1.61	1–7
	IM stimulation	4.81	1.59	1–6
	IM achievement	5.07	1.42	1–6
	Integrated Reg	4.19	1.74	1–6
	Identified Reg	4.78	1.41	1–6
	Introjected Reg	2.72	1.53	1–6
	External Reg	1.86	1.20	1–6
	Amotivation	2.08	1.30	1–6
Importance of Physical Education	PE Importance	2.77	0.79	1–4
Physical activity intentions	Intentions	3.70	0.91	1–5
Habitual physical activity	PA Level	9.85	5.21	1–5

M = Mean; SD = Standard deviation; R = Range.

**Table 2:** Correlations among study variables.

Dimension	1	2	3	4	5	6	7	8	9	10	11	12	13	14	15	16
1.Teach Control	-	−0.08	−0.02	0.09	−0.11 *	0.08	−0.01	0.00	0.08	0.04	0.35 **	0.45 **	0.40 **	−0.04	0.04	0.06
2.Competence	-	-	0.59 **	0.42 **	0.54 **	0.52 **	0.59 **	0.57 **	0.55 **	0.55 **	0.13 **	−0.14 **	−0.17 **	0.35 **	0.53 **	0.42 **
3. Autonomy	-	-	-	0.46 **	0.42 **	0.46 **	0.50 **	0.45 **	0.45 **	0.46 **	0.17 **	−0.05	−0.09	0.30 **	0.41 **	0.33 **
4. Relatedness	-	-	-	-	0.33 **	0.39 **	0.40 **	0.42 **	0.42 **	0.42 **	0.27 **	0.12 *	0.10	0.32 **	0.35 **	0.20 **
5. IM general	-	-	-	-	-	0.73 **	0.80 **	0.70 **	0.64 **	0.66 **	0.12 *	−0.15 **	−0.28 **	00.43 **	0.66 **	0.44 **
6. IM knowledge	-	-	-	-	-	-	0.84 **	0.79 **	0.74 **	0.78 **	0.32 **	0.04	−0.16 **	0.54 **	0.65 **	0.50 **
7. IM stimulation	-	-	-	-	-	-	-	0.85 **	0.75 **	0.79 **	0.27 **	−0.04	−0.18 **	0.46 **	0.68 **	0.52 **
8. IM achievement	-	-	-	-	-	-	-	-	0.75 **	0.81 **	0.31 **	−0.05	−0.20 **	0.48 **	0.66 **	0.49 **
9. Integrated Reg	-	-	-	-	-	-	-	-	-	0.77 **	0.42 **	0.06	−0.10 *	0.45 **	0.68 **	0.57 **
10. Identified Reg	-	-	-	-	-	-	-	-	-	-	0.40 **	0.03	−0.16 **	0.54 **	0.66 **	0.50 **
11. Introjected Reg	-	-	-	-	-	-	-	-	-	-	-	0.55 **	0.30 **	0.19 **	0.27 **	0.21 **
12. External Reg	-	-	-	-	-	-	-	-	-	-	-	-	0.58 **	0.04	−0.03	0.01
13. Amotivation	-	-	-	-	-	-	-	-	-	-	-	-	-	−0.08	−0.20 **	−0.10 *
14. PE Importance	-	-	-	-	-	-	-	-	-	-	-	-	-	-	0.53**	0.33 **
15. Intentions	-	-	-	-	-	-	-	-	-	-	-	-	-	-	-	0.66 **
16. PA Level	-	-	-	-	-	-	-	-	-	-	-	-	-	-	-	-

** *p* < 0.001 * *p* < 0.005; IM = Intrinsic motivation; Reg = Regulation; PE = Physical Education; PA = Physical activity.

**Table 3:** Means and standard deviations for the variables in each cluster for each sample.

Dimension	Sample 1				Sample 2				Sample Total			
	Cluster 1 (*n* = 83)		Cluster 2 (*n* = 125)		Cluster 1 (*n* = 57)		Cluster 2 (*n* = 151)		Cluster 1 (*n* = 239)		Cluster 2 (*n* = 177)	
	*M*	*SD*	*M*	*SD*	*M*	*SD*	*M*	*SD*	*M*	*SD*	*M*	*SD*
IM general	6.16	0.81	4.23	1.27	4.30	1.50	6.34	0.72	6.22	0.80	4.27	1.39
IM knowledge	5.43	1.16	3.43	1.27	2.96	1.19	5.64	1.00	5.53	1.07	3.27	1.28
IM stimulation	5.68	1.04	3.60	1.17	3.13	1.30	5.95	0.78	5.83	0.91	3.43	1.22
IM achieve	5.90	0.88	3.92	1.08	3.69	1.10	6.09	0.73	6.00	0.80	3.82	1.09
Integrated Reg	5.14	1.24	3.07	1.16	2.29	1.04	5.31	1.35	5.23	1.31	2.78	1.18
Identified Reg	5.64	0.84	3.75	1.05	3.36	1.17	5.69	0.87	5.65	0.87	3.60	1.10
Introjected Reg	3.29	1.59	2.87	1.31	1.51	0.76	2.75	1.64	2.90	1.64	2.48	1.33
External Reg	2.29	1.43	2.42	1.36	1.24	0.76	1.38	0.63	1.69	1.08	2.08	1.33
Amotivation	2.89	1.23	3.19	1.12	1.11	0.18	1.08	0.16	1.71	1.13	2.58	1.34

Note: IM = Intrinsic motivation; R = Regulation; M = Mean; SD = Standard deviation.

**Table 4:** Multivariate analysis.

Variables	Cluster 1 Self-Determinated (*n* = 239)		Cluster 2 Less Self-Determinated (*n* = 177)			
	*M*	*DT*	*M*	*DT*	*F*	ƞ^2^
1. Controlling Style	2.50	1.15	2.76	1.21	5.22 *	0.01
2. Competence	4.85	1.03	3.69	1.03	130.35 **	0.24
3. Autonomy	4.37	1.01	3.53	0.99	72.21 **	0.15
4. Relatedness	4.00	0.99	3.30	0.95	51.67 **	0.11
5. PE Importance	3.03	0.69	2.42	0.77	72.21 **	0.15
6. PA Intentions	4.18	0.67	3.05	0.78	249.09 **	0.38
7. PA Levels	11.77	4.84	7.26	4.53	93.31 **	0.19
Wilk’s Λ					0.56	
Multivariate F					45.50	

* *p* < 0.05; ** *p* < 0.01; M = Mean; SD = Standard deviation; PE = Physical Education; PA = Physical activity.
